# Tracking the evolution of sex chromosome systems in Melanoplinae grasshoppers through chromosomal mapping of repetitive DNA sequences

**DOI:** 10.1186/1471-2148-13-167

**Published:** 2013-08-09

**Authors:** Octavio M Palacios-Gimenez, Elio R Castillo, Dardo A Martí, Diogo C Cabral-de-Mello

**Affiliations:** 1 UNESP - Univ Estadual Paulista, Instituto de Biociências/IB, Depto. de Biologia, Rio Claro/SP, Brazil; 2 Laboratorio de Genética Evolutiva, IBS, Facultad de Ciencias Exactas, Químicas y Naturales, Universidad Nacional de Misiones, Posadas 3300, Argentina

## Abstract

**Background:**

The accumulation of repetitive DNA during sex chromosome differentiation is a common feature of many eukaryotes and becomes more evident after recombination has been restricted or abolished. The accumulated repetitive sequences include multigene families, microsatellites, satellite DNAs and mobile elements, all of which are important for the structural remodeling of heterochromatin. In grasshoppers, derived sex chromosome systems, such as neo-XY♂/XX♀ and neo-X_1_X_2_Y♂/X_1_X_1_X_2_X_2_♀, are frequently observed in the Melanoplinae subfamily. However, no studies concerning the evolution of sex chromosomes in Melanoplinae have addressed the role of the repetitive DNA sequences. To further investigate the evolution of sex chromosomes in grasshoppers, we used classical cytogenetic and FISH analyses to examine the repetitive DNA sequences in six phylogenetically related Melanoplinae species with X0♂/XX♀, neo-XY♂/XX♀ and neo-X_1_X_2_Y♂/X_1_X_1_X_2_X_2_♀ sex chromosome systems.

**Results:**

Our data indicate a non-spreading of heterochromatic blocks and pool of repetitive DNAs (*C*_*0*_*t*-1 DNA) in the sex chromosomes; however, the spreading of multigene families among the neo-sex chromosomes of *Eurotettix* and *Dichromatos* was remarkable, particularly for 5S rDNA. In autosomes, FISH mapping of multigene families revealed distinct patterns of chromosomal organization at the intra- and intergenomic levels.

**Conclusions:**

These results suggest a common origin and subsequent differential accumulation of repetitive DNAs in the sex chromosomes of *Dichromatos* and an independent origin of the sex chromosomes of the neo-XY and neo-X_1_X_2_Y systems. Our data indicate a possible role for repetitive DNAs in the diversification of sex chromosome systems in grasshoppers.

## Background

For more than a century, the evolution of the sex chromosomes and the genetics of sex determination have been the source of some of the most intriguing questions in evolutionary biology and have been the focus of many genetic and cytological studies (see for example [1-12]). Sex chromosomes evolve from a pair of homologous autosomes [13], and the restriction or absence of recombination and the further accumulation of repetitive sequences on chromosomes Y or W are important events in the differentiation of these elements [14-16].

Based on evidence obtained from molecular studies in different taxa, DNA sequence restructuring occurs within new sex chromosome regions (Y or W) during the early evolution of the sex chromosomes, and this process involves modifications to the chromatin structure and the insertion of repetitive DNA sequences. These morphological and genetic changes are consistent with the abolition of recombination, which precedes the genetic degeneration of neo-Y or neo-W chromosomes with unknown fates [1,15-21].

Among the inserted repetitive DNA sequences, a remarkable preponderance of mobile elements, satellite DNAs, microsatellites and multigene families, which can remodel euchromatic structures into heterochromatin, has been observed [17,22-25]. Non-recombining regions of the Y chromosome containing accumulated repetitive DNAs have been well documented in, for example, mammalian species [11,26] and *Drosophila melanogaster*[27], in which the sex chromosome systems are evolutionarily ancient [8,28]. The accumulation of repetitive sequences, even in young sex chromosomes, has also been observed in other organisms, such as *Drosophila miranda*[19], *Silene latifolia*[29-31] and *Rumex acetosa*[23].

Grasshopper species are characterized by a high frequency of 2n=23♂/24♀ karyotypes comprising acro-telocentric chromosomes and X0♂/XX♀ sex chromosome determination system. According to White [32] and Hewitt [4], this karyotype is considered atavistic, at least for Caelifera. Although grasshoppers within Acrididae have this form of karyotypic stability and X0♂/XX♀ sex chromosome system, the Melanoplinae subfamily shows an unusually high frequency of derived neo-sex chromosome systems, which have been observed in at least 50 species [33-35]. This sex chromosome variability primarily reflects the occurrence of Robertsonian fusions (Rb-fusions), which generate complex neo-XY♂/XX♀ and neo-X_1_X_2_Y♂/X_1_X_1_X_2_X_2_♀ sex chromosome systems [32,35-37].

In contrast with other insect orders such as Lepidoptera [38-41] and Diptera [6,7,21] in which the evolution of the sex chromosomes has been studied by mapping distinct classes of DNAs, there is a complete lack of knowledge at the molecular level concerning the evolution of the neo-sex chromosomes in grasshoppers and the mechanisms that underlie the degeneration of the neo-Y chromosome. The great diversity of the sex chromosome systems observed in Melanoplinae suggests that this group represents an excellent experimental model to analyze any changes in patterns of linked gene groups within the sex chromosomes. With the aim of a better understanding of the evolution of sex chromosomes in grasshoppers we used classical cytogenetic techniques and fluorescence *in situ* hybridization (FISH) to analyze five multigene families, telomeric repeats and repetitive DNA fractions (*C*_*0*_*t*-1 DNA fraction) in six phylogenetically related Melanoplinae species: *Chlorus vittatus* and *Ch. chiquitensis*; *Eurotettix minor* and *E. brevicerci*; and *Dichromatos lilloanus* and *D. schrottkyi*[42,43]. These species present different sex chromosomes, including X0, neo-XY and neo-X_1_X_2_Y in males ([33,34], this work). We focused mainly on the dynamics of repetitive DNA incorporation into new sex chromosomes as an evolutionary force that contributes to the chromosomal diversification of this group, and we examined the evidence for independent or common origins of the neo-sex chromosome systems in the analyzed species.

## Results

### Meiosis and karyotypes

**Figure 1:**
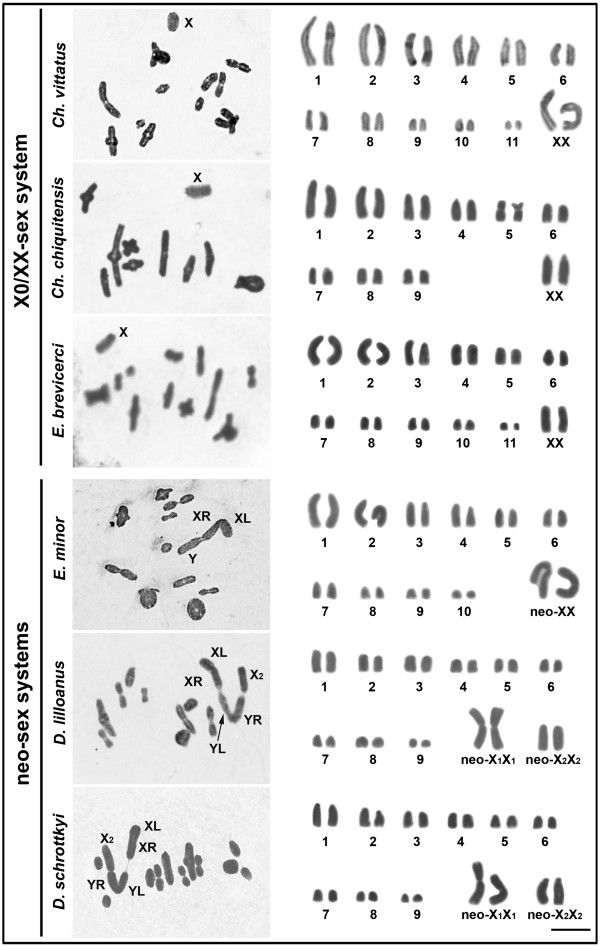
**Conventional staining of male metaphase I (left panel) and female mitotic karyotypes (right panel).** The sex-chromosome system types and the species names are shown directly in the figure. The sex chromosomes and chromosome arms of the neo-sex chromosomes involved in Rb-fusions are indicated. XL: arm derived from the original X chromosome fused to an autosome; XR: autosomal arm of the neo-X that shares homology with the neo-Y; YL: arm that shares homology with the XR arm; YR: arm that shares homology with the neo-X_2_ chromosome. Bar = 5 μm.

Different diploid numbers were observed in the six species studied: 2n=23♂/24♀ in *Chlorus vittatus* and *Eurotettix brevicerci*, 2n=19♂/20♀ in *Ch. chiquitensis*, 2n=22♂/22♀ in *E. minor* and 2n=21♂/22♀ in *Dichromatos lilloanus* and *D. schrottkyi* (Figure 1; Table 1). The autosomes were, in general, acro-telocentric; however, in *Ch. chiquitensis*, pair 5 was submetacentric. Three types of sex chromosome systems were observed: X0♂/XX♀ in *Ch. vittatus*, *Ch. chiquitensis* and *E. brevicerci*; neo-XY♂/XX♀ in *E. minor* and neo-X_1_X_2_Y♂/X_1_X_1_X_2_X_2_♀ in *D. lilloanus* and *D. schrottkyi* (Figure 1; Table 1).

**Table 1:** **Species, locality, number of males and females (M/F), diploid numbers and chromosomal positions of multigene families in grasshoppers from the *Chlorus*, *Eurotettix *and *Dichromatos *genera studied in this paper**

**Species**	**Locality**	**Number of individuals (M/F)**	**2n**	**18S rDNA**	**H3 histone gene**	**5S rDNA**	**U1 snDNA**	**U2 snDNA**
*Ch. vittatus*	Parque Nacional Ybycuí (Paraguay)	15/2	♂23/X0	3 pc; 6 d	7 i	3 i; 4 d; 6 i	4 d	1 i; 2 i; 9 i
			♀24/XX					
*Ch. chquitensis*	Corumba (Brazil)	9/2	♂19/X0	X pc	7 pc	6 i	4 d	1 i; 2 i
			♀20/XX					
*E. brevicerci*	Botucatu (Brazil)	15/9	♂23/X0	X pc; 3 pc	7 pr	3 i	4 d	1 i; 9 pc*
			♀24/XX					
*E. minor*	Paraguarí (Paraguay)	16/4	♂22/XY	3 pc	5 d	3 pc; 5 pr; XR i	4 d; XR i;Y i	1 i; 2 i
			♀22/XX					
	Atyra (Paraguay)	2/0						
	Altos (Paraguay)	1/0						
	Parque Nacional Ybycuí (Paraguay)	10/1						
	Ybycuí (Paraguay)	7/0						
*D. lilloanus*	Reserva Provincial Yaguaroundí (Argentina) Eldorado (Argentina)	30/35	♂21/X_1_X_2_Y	X_1_ pc; 5 pr	1-9 pc; X_1_ pc; X_2_ pc	3 i; YL pr, i, d; YR pr	2 i	1 i; 6 pr; YL pr
		0/2	♀22/X_1_X_1_X_2_X_2_					
*D. schrottkyi*	Eldorado (Argentina)	2/4	♂21/X_1_X_2_Y	X_1_ pc; 4 pr	5 pr	2 i; YL i; YR pr, i	3 d	1 i; 5 pr *
			♀22/X_1_X_1_X_2_X_2_					

*pc*=pericentromeric; *pr*=proximal; *i*=intersticial; *d*=distal. * indicate the occurrence of heteromorphism.

The X sex chromosome in the X0♂/XX♀ system was acro-telocentric, showing negative heteropycnotic behavior during metaphase I and variability in size among the species (Figure 1). In *E. minor*, the neo-XY♂/XX♀ sex pair was formed by a metacentric neo-X, the product of Rb-fusion between the ancestral X and an autosome, whose homologue has become a telocentric neo-Y. The neo-sex chromosomes showed distal contact during metaphase I, and adopted the typical L-shaped configuration (Figure 1). Finally, in the *Dichromatos* species, neo-sex chromosomes were formed from the metacentric neo-X_1_, the acro-telocentric neo-X_2_ and the metacentric neo-Y, being the neo-Y chromosome the largest element. At metaphase I, the neo-sex chromosomes were observed in the typical convergent orientation of a Robertsonian trivalent, with the XR arm distally associated with the YL arm of the neo-Y chromosome and the YR arm of the neo-Y chromosome distally associated with the neo-X_2_ chromosome (Figure 1).

### Heterochromatin, C_0_t-1 DNA and telomeric mapping

**Figure 2:**
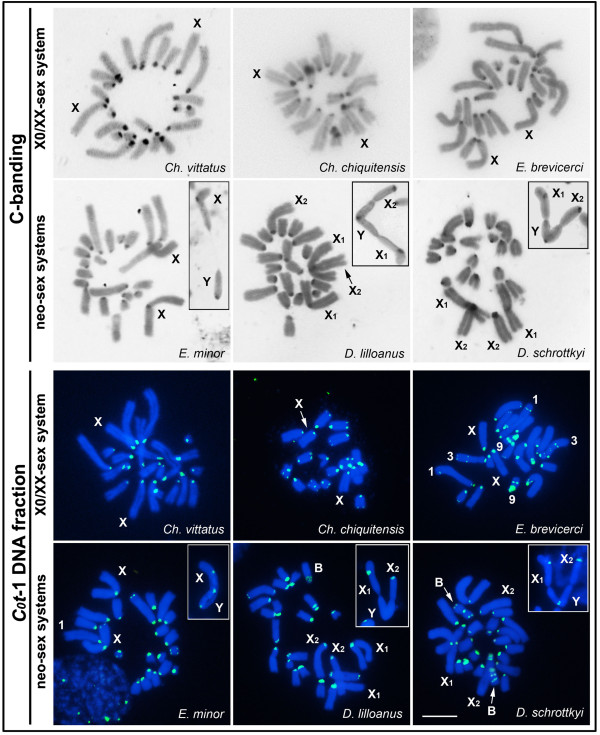
**C-banding and *C***_***0***_***t*-1 DNA fractions in female mitotic chromosomes.** The species names and the types of sex chromosome determination systems are indicated in each figure. Inserts show the locations of C-banding and *C*_*0*_*t*-1 DNA fractions in the neo-sex chromosomes during meiosis. Note the absence of the *C*_*0*_*t*-1 DNA fraction in the neo-Y chromosome of *D. lilloanus.* Bar = 5 μm.

In all of the species analyzed here, C-positive blocks were observed in the pericentromeric region of all complements, including the sex chromosomes (Figure 2). These C-positive regions were labeled by the *C*_*0*_*t*-1 DNA fractions obtained from each species, except the pericentromeric region of the neo-Y chromosome in *D. lilloanus*. Additionally, terminal blocks were detected in the *Ch. chiquitensis*, *Eurotettix* and *D. lilloanus* chromosomes. In *E. brevicerci*, interstitial blocks were also observed in pairs 1, 3 and 9 (Figure 2). In the *Dichromatos* species, the specimens used to perform the FISH analysis with the *C*_*0*_*t*-1 DNA probes carried B chromosomes that presented pericentromeric, interstitial or terminal blocks (Figure 2).

**Figure 3:**
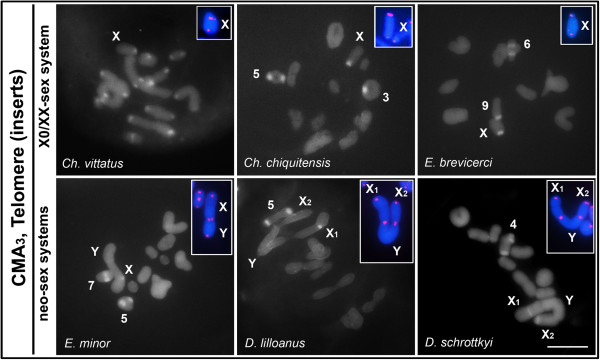
**CMA**_**3 **_**fluorochrome staining and FISH with a telomeric probe (insert) in male meiotic cells.** The species names and sex chromosome system types are indicated in each figure. Additionally, autosomes with CMA_3_-positive blocks are indicated. Note the absence of interstitial telomeric sites in the neo-sex chromosomes produced by Rb-fusions. Bar = 5 μm.

The CMA_3_/DAPI fluorochrome staining revealed homogeneous DAPI staining (results not shown) and distinct patterns of G+C-rich blocks (CMA_3_ positive) as follows: *Ch. vittatus*, all pericentromeric regions; *Ch. chiquitensis*, pericentromeric regions of pairs 3, 5 and the X chromosome; *E. brevicerci*, interstitial region of pair 6 and pericentromeric regions of the X chromosome and pair 9 (heteromorphic); *E. minor*, pericentromeric region of pair 5 and the neo-X chromosome and the distal region of pair 7; *D. lilloanus*, pericentromeric regions of pair 5 and the neo-X_1_ chromosome; *D. schrottkyi*, pericentromeric regions of pair 4 and the neo-X_1_ and neo-X_2_ chromosomes (Figure 3).

In all species with X0♂/XX♀ sex system and those with Rb-fusion-derived sex chromosomes (neo-XY♂/XX♀, neo-X_1_X_2_Y♂/X_1_X_1_X_2_X_2_♀), only terminal sites were observed with a telomeric probe in both the autosomes (result not shown) and the sex chromosomes (Figure 3, insets).

### Cytogenetic mapping of multigene families

**Figure 4:**
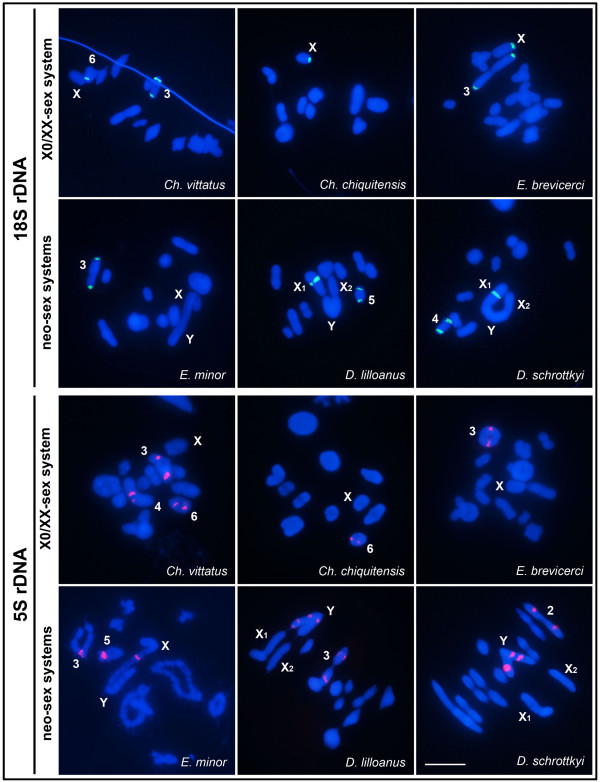
**FISH with 18S and 5S rDNA probes in meiotic cells from males.** The probe, type of sex-chromosome system and name of species are indicated in each figure. Chromosomes with positive signals and sex chromosomes are indicated. Note the presence of pericentromeric sites for 18S rDNA on the X-chromosomes of *Ch. chiquitensis* and *E. brevicerci* and on the neo-X_1_ chromosomes of *D. lilloanus* and *D. schrottkyi*, as well as the 5S rDNA on the sex bivalent neo-XY chromosome of *E. minor* and in multiple sites on the neo-Y chromosome in *D. lilloanus* and *D. schrottkyi*. Bar = 5 μm.

FISH analysis with an 18S rDNA probe revealed signals in two autosomal pairs in *Ch. vittatus,* the X chromosome of *Ch. chiquitensis* and one pair of autosomes in *E. brevicerci* and *E. minor*; an additional cluster in the X chromosome of *E. brevicerci* was also observed (Table 1; Figure 4). In the *Dichromatos* species, signals were detected in the neo-X_1_ chromosome and in one autosomal pair (*D. lilloanus* pair 5 and *D. schrottkyi* pair 4) (Table 1; Figure 4).

Hybridization signals of the 5S rDNA probe were observed in three autosomal pairs of *Ch. vittatus*, but only in one pair of autosomes in *Ch. chiquitensis* and *E. brevicerci* (Table 1; Figure 4). *Eurotettix minor* showed clusters of the 5S rDNA genes in two autosomal pairs and in the XR arm of the neo-X chromosome, whereas *D. lillonaus* and *D. schrottkyi* each showed one cluster in a pair of autosomes and multiple 5S rDNA sites in the neo-Y chromosome (Table 1; Figure 4).

**Figure 5:**
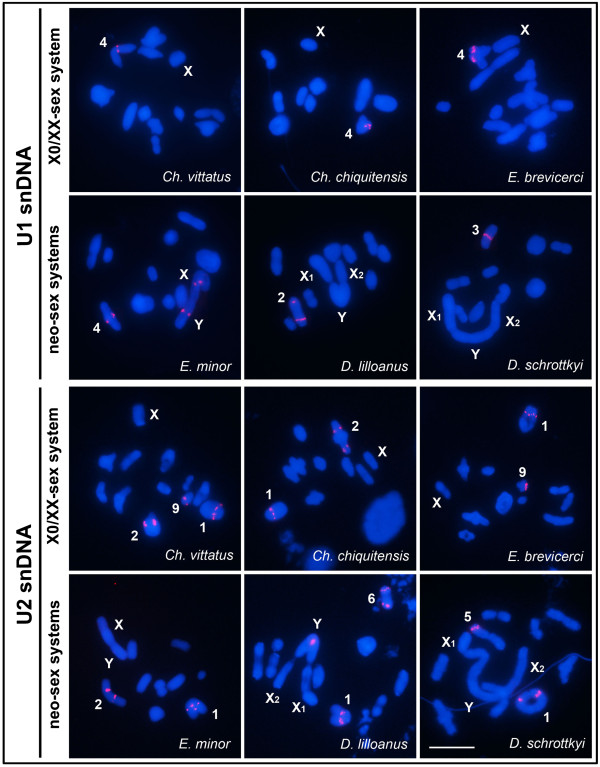
**Chromosomal mapping of the U1 and U2 snRNA genes in meiotic cells from males.** The probe type, sex chromosome system and name of species are shown for each cell. Chromosomes with positive hybridization signals and sex chromosomes are indicated in the images. Note the presence of U1 snDNA clusters in the interstitial region of the neo-XY chromosomes of *E. minor*, and the U2 snDNA clusters in the proximal region of the neo-Y chromosome of *D. lilloanus*. Bar = 5 μm.

In four species, *Ch. vittatus*, *Ch. chiquitensis*, *E. brevicerci* and *E. minor*, the U1 snRNA gene was distally located in pair 4. Additionally, U1 snRNA was present at interstitial sites in the XR and neo-Y chromosomes of *E. minor*. *Dichromatos lilloanus* and *D. schrottkyi* showed U1 snDNA clusters only in one bivalent (Table 1; Figure 5). U2 snDNA clusters were located interstitially in two autosomal pairs in the *Chlorus* species, and in *Ch. vittatus,* U2 snDNA was detected in an additional autosomal pair. In the *Eurotettix* species, these sequences were observed in two autosomal pairs. *Dichromatos* showed hybridization signals in two autosomal pairs; however, this gene cluster was also located on the YL arm in *D. lilloanus* (Table 1; Figure 5).

**Figure 6:**
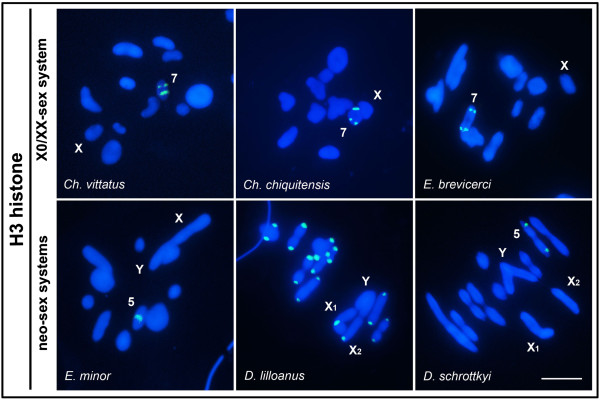
**FISH for the histone H3 gene in male meiotic cells.** Divergent sex chromosomes, names of species and the chromosomes with hybridization signals are indicated in the figure. Remarkably, the histone H3 gene cluster was found in pericentromeric regions of all chromosomes, except in the neo-Y in *D. lilloanus*. Bar = 5 μm.

Finally, FISH analysis of histone H3 revealed conserved hybridization signals in pair 7 of *Ch. vittatus*, *Ch. chiquitensis* and *E. brevicerci*. *Eurotettix minor* and *D. schrottkyi* presented the histone H3 cluster in the distal and interstitial regions of pair 5, respectively; whereas in *D. lilloanus*, this gene was spread throughout the pericentromeric regions of all chromosomes, except for the neo-Y chromosome (Table 1; Figure 6).

**Figure 7:**
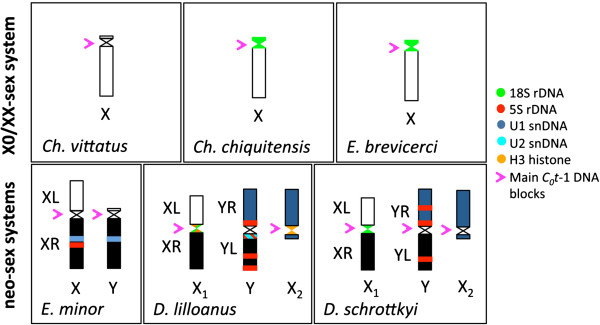
**FISH signals with six probes in the sex chromosomes of all the species analyzed in this study.** The probes and its position on the sex chromosomes are shown. Colors on the sex chromosomal arms represent the ancestral regions of homology.

The FISH results showing the chromosomal locations of the multigene families are summarized in Table 1, and the FISH results for the sex chromosomes are summarized in Figure 7, except for those obtained using the telomeric probe.

## Discussion

### General organization of repetitive DNAs in autosomes

The general distribution patterns of the C-positive blocks found in the studied species were similar to those reported for other grasshopper species and occurred as pericentromeric blocks in the autosomal complements [34,44,45]. However, other repetitive DNA rich regions were detected using the *C*_*0*_*t*-1 DNA fraction, including telomeres and interstitial areas.

For the multigene families, intra- and intergenomic variability were observed for the distinct sequences and species. Our findings revealed remarkable variability in the number and location of major rDNA genes; this is consistent with previous studies in which similar patterns were observed in grasshopper species [45-47] and in other insects, such as Lepidoptera [48], Coleoptera [49] and Heteroptera [50]. The variability for 5S rDNA also reflects common patterns seen in grasshoppers [47]. In contrast with the rDNAs, the Melanoplinae species analyzed here showed less variability in the U1 snRNA genes; this stability of the U1 snDNA clusters has been previously documented in other biological models, such as in cichlid fishes [51]. Although an additional U snRNA gene, U2 snRNA, showed more variability than U1 snRNA, it was also conserved in the interstitial position of pair 1, potentially reflecting the ancestral placement in these species.

According to Cabrero et al. [52], the occurrence of one autosomal cluster of histone H3 genes represents the ancestral placement for Acrididae. This location was observed in our study for five of the species analyzed. However, it is possible that the unusual dispersion observed for the histone H3 genes in *D. lilloanus*, also observed for example in *Abracris flavolineata*[53], could be the result of multiple mechanisms, such as association with mobile elements, ectopic recombination or extrachromosomal circular DNA (eccDNA), as has been postulated for rDNAs [46,54-56].

### Diversification of the sex chromosomes

As we mentioned above, the organization of different repetitive DNA sequences has been described in grasshoppers, mainly for multigene families [46,47,52]. However, there are no records of studies focusing on the possible role of such genomic elements in the diversification of sex chromosomes.

C-positive blocks in the pericentromeric regions observed in the three different sex chromosome systems indicate non-spreading of heterochromatic segments after their origin. Additionally, the mapping of *C*_*0*_*t*-1 DNA fractions reinforced the non-massive spreading of repetitive DNA pools in these sex chromosomes, which contrasts with the repetitive DNA accumulation expected on sex chromosomes after recombination restriction [19,25,31,57]. An alternative hypothesis is that these chromosomes possess variable repetitive DNAs not isolated in the *C*_*0*_*t*-1 DNA fraction.

For *E. minor*, *D. schrottkyi* and *D. lilloanus*, the mapping of the *C*_*0*_*t*-1 DNA fraction suggested different evolutionary scenarios for the divergence of the neo-Y sex chromosomes. The results could be interpreted as evidence of the loss of selection pressure in the non-recombining regions during their differentiation, leading to a high rate of genetic diversification in the neo-Y chromosome. In the *D. lilloanus* neo-Y chromosome, we observed the absence of a *C*_*0*_*t*-1 DNA block compared with the *E. minor* and *D. schrottkyi* neo-Y chromosomes. Different accumulation/diversification patterns of repetitive DNAs in sex chromosomes were also documented for example in plants from the *Rumex* genus [23] and Parodontidae fish [58].

Considering the presence of all of the multigene families mapped in the sex chromosomes, we propose that these sequences could be involved in the diversification of the sex-chromosome determining mechanisms found in Melanoplinae. The 18S rDNA mapping results indicate the independent evolution of the neo-XY and neo-X_1_X_2_Y sex systems in the related genera *Eurotettix* and *Dichromatos*, due to the absence and presence of this marker in the X chromosomes, respectively. However, we could not rule out the possibility of transposition in these derived sex chromosomes. The noticeable accumulation of 5S rDNA in the XR arm of *E. minor* and the neo-Y chromosome of *D. lilloanus* and *D. schrottkyi* could initially be attributed to Rb-fusion X-A and also due to the absence of recombination between sex chromosomes, with the gene cluster localized on the autosome involved in the rearrangement. Moreover, the presence of multiple sites containing 5S rDNA on the neo-Y chromosome of *D. lilloanus* and *D. schrottkyi* suggests the strong accumulation of these sequences after chromosomal rearrangement or the potential action of intrachromosomal recombination, followed by amplification and transposition. The multiple sites observed for these sequences could make this region less likely to undergo recombination and allow it to play an important role in chromatin remodeling, as has been observed for other repetitive DNAs. The rDNA locus, located on sex chromosomes in salmonid fishes, for example, has been suggested to be involved in the restriction of crossing-over near the sex-determining locus [59].

The U1 snRNA gene did not show a strong relationship with sex chromosome diversification, occurring only in the neo-XY chromosome of *E. minor*; this result supports the existence of divergent evolutionary pathways from the *Dichromatos* neo-sex chromosomes. For *D. lilloanus,* the presence of U2 snDNA in the neo-Y chromosome demonstrates the diversification of this chromosome relative to the other congeneric species, *D. schrottkyi*. Although the histone H3 genes were present in the neo-X_1_ and neo-X_2_ chromosomes of *D. lilloanus*, this sequence was not apparently consistent with sex chromosome diversification; this phenomenon could be associated with the intrinsic mechanism of histone H3 dispersion in the *D. lilloanus* genome after the divergence of the *Dichromatos* species.

Phylogenetic analyses suggest that *Chlorus*, *Eurotettix* and *Dichromatos* are monophyletic groups with uncertain evolutionary relationships to the rest of the Dichroplini tribe [42,43]. Considering the morphological characteristics of these species with all brachypterous species, and consequently low vagility, it is possible that the neo-sex chromosome systems might have played a significant role in the divergence and isolation between populations, leading to the restriction of gene flow and speciation. After isolation, the sex chromosomes could undergo molecular differentiation, as observed for the species studied here. Similar models of phenotypic divergence, reproductive isolation and speciation through neo-sex chromosomes have been proposed, for example, for closely related species of fishes [12].

Notably, neo-sex chromosome systems derived from autosome-sex chromosome fusion have been frequently reported in animals [3,12,60-62]. Such rearrangement results in specific intrinsic properties, such as recombination-free regions, due to chiasmata shifts that lead to low intra-chromosomal recombination between involved chromosomes, and the reduction of linkage groups, resulting in lower rates of inter-chromosomal recombination [3,15,21]. According to Charlesworth et al. [20], these factors create strong linkage between the genes on evolving sex chromosomes, which is favorable in the heterogametic sex. These mechanisms might potentially be involved in sex chromosome diversification among Melanoplinae grasshoppers undergoing Rb-fusions that result in reduced chromosome numbers. Indeed, we demonstrated in this study that the presence of telomeric sequences occurred only in current telomeres, which confirms a previous hypothesis that Rb-fusions [32] originate from double chromosome breaks with the loss of telomeric sequences. Although we cannot rule out completely the occurrence of interstitial telomeric sites not detected by FISH.

## Conclusions

Different organization of repetitive sequences in the sex chromosomes indicates independent diversification of the sex chromosome systems in Melanoplinae grasshoppers of the *Chlorus*, *Eurotettix* and *Dichromatos* genera. However, the localization of 18S and 5S rDNA on the neo-X_1_ and neo-Y chromosomes of *Dichromatos* species suggests that the neo-X_1_X_2_Y sex determination systems share a common origin, but these chromosomes have also undergone distinct modifications that led to their differentiation. In addition, the presence of structural genes (like 5S rRNA, U1 snRNA and U2 snRNA) mapped to the neo-Y chromosome of *E. minor* and *Dichromatos* species would prevent the complete degeneration and loss of these chromosomes (X0 reversion). The results presented in this paper provide an initial characterization of the derived sex chromosomes in grasshoppers at a molecular level, focusing on the presence of repetitive DNA sequences. To obtain a more detailed picture of sex chromosome evolution in grasshoppers, future studies should be performed using cross-species chromosome painting and the isolation of different repetitive DNAs, such as transposable elements and satellite DNAs.

## Methods

### Animals, DNA samples and chromosome spreading

Male and female adult grasshoppers from the species *Chlorus vittatus*, *Ch. chiquitensis*, *Eurotettix brevicerci*, *E. minor*, *Dichromatos lilloanus* and *D. schrottkyi* were sampled from distinct localities in Paraguay, Argentina and Brazil (Table 1). Male testes were fixed in a 3:1 ethanol: acetic acid solution, and female gastric caeca were removed and fixed as described by Castillo et al. [63]. All specimens were stored in 100% ethanol until subsequent DNA extraction.

We used conventional staining with 5% Giemsa to visualize the general chromosomal characteristics present in the individuals of each species. C-banding was performed according to Sumner [64], and fluorochrome staining (CMA_3_/DA/DAPI) was performed according to Schweizer et al. [65]. Genomic DNA extraction was performed using the phenol-chloroform protocol [66].

The nomenclature proposed by White [3] was used to describe the neo-sex chromosome arms in simple neo-XY systems; the arms of neo-X chromosomes were designated XL, which is the ancestral X, and XR, which shares homology with the neo-Y. In multiple neo-X_1_X_2_Y systems, the neo-X_1_ chromosome was designated as described for the neo-XY type; the metacentric neo-Y chromosome is formed from the YL and YR arms, which share homology with the XR and neo-X_2_ chromosome, respectively.

### Isolation of multigene families and telomeric repeats

The partial sequences of the 5S rRNA and histone H3 genes were amplified by polymerase chain reaction (PCR) using genomic DNA obtained from *Abracris flavolineata* and the primers described by Loreto et al. [67] and Cabral-de-Mello et al. [68] for 5S rDNA and Colgan et al. [69] for histone H3. The sequences for the U snDNAs were obtained from the *Rhammatocerus brasiliensis* genome using primers described by Cabral-de-Mello et al. [51] for U1 snDNA and Bueno et al. [53] for U2 snDNA. The amplified fragments were sequenced and deposited in GenBank under the accession numbers KC936996 (5S rDNA), KC896792 (histone H3 gene), KC896793 (U1 snDNA) and KC896794 (U2 snDNA).

The 18S rDNA sequence was obtained from a cloned fragment previously isolated from the *Dichotomius semisquamosus* genome (GenBank accession number GQ443313, Cabral-de Mello et al. [68]), and the telomeric probes were obtained by PCR using the complementary primers (TTAGG)_5_ and (CCTAA)_5_[70].

### C_0_t-1 DNA isolation

Repetitive DNA-enriched samples from each species were obtained based on the renaturation kinetics of *C*_*0*_*t*-1 DNA (DNA enriched for highly and moderately repetitive DNA sequences), according to the protocol described by Zwick et al. [71] with modifications [68]. Briefly, the DNA samples (200 μL of 100–500-ng/μL genomic DNA in 0.3 M NaCl) were digested with deoxyribonuclease I (Sigma, St Louis, MO, USA) at 0.01 U/μL for 80 to 105 sec, depending on the sample concentration, and the fragmented DNA was separated using 1% agarose gel electrophoresis. The expected DNA fragments ranged in size from 100 to 1,000 base pairs (bp). For each species, 50 μL samples of the fragmented DNA were denatured at 95°C for 10 min, placed on ice for 10 sec and transferred to a 65°C water bath to reanneal for 25 min. Subsequently, the samples were incubated at 37°C for 8 min with 1 U of S1 nuclease to digest the single-stranded DNA. The DNA was purified and extracted using a traditional phenol-chloroform protocol [66].

### *Fluorescence* in situ *hybridization*

The plasmid containing the 18S rRNA gene, the PCR products from the histone H3 gene and the *C*_*0*_*t*-1 DNA fraction were labeled by nick translation using biotin-14-dATP (Invitrogen, San Diego, CA, USA). The 5S rDNA, U snDNAs (U1, U2) and telomeric probes were PCR labeled with digoxigenin-11-dUTP (Roche, Mannheim, Germany).

Single- or two-color FISH was performed according to Pinkel et al. [72], with modifications [68] using distinct mitotic and meiotic cells. Although some two-color FISH assays were performed, the same metaphase is shown separately for each probe. Probes labeled with digoxigenin-11-dUTP were detected using anti-digoxigenin-rhodamine (Roche), and probes labeled with biotin-14-dATP were detected using streptavidin, alexa fluor 488 conjugate (Invitrogen). The preparations were counterstained using 4′, 6-diamidine-2′-phenylindole dihydrochloride (DAPI) and mounted using Vectashield (Vector, Burlingame, CA, USA). The chromosomes and FISH signals were observed using an Olympus microscope BX61 equipped with a fluorescence lamp and appropriate filters. The photographs were recorded using a DP70 cooled digital camera. The images were merged and optimized for brightness and contrast using Adobe Photoshop CS2 software.

## Abbreviations

2n: diploid number; Bp: Base pairs; CMA3: Chromomycin A_3_; C0t: *C*_*0*_ is the initial concentration of single-stranded DNA in mol/l and *t* is the reannealing time in seconds; DA: Distamicyn; DAPI: 4′, 6-Diamidino-2-phenylindole; FISH: Fluorescence *in situ* hybridization; PCR: Polymerase chain reaction; Rb-fusion: Robertsonian fusion; rDNA: Ribosomal DNA; rRNA: Ribosomal RNA; snRNA: Small nuclear RNA.

## Competing interests

The authors declare that they have no competing interests.

## Authors’ contributions

OMPG conducted the chromosome preparations and the molecular cytogenetic experiments, interpreted the data, and drafted of the manuscript. ERC and DAM interpreted the data and drafted the manuscript. DCCM conceived the study, participated in its design and coordination, interpreted the data and assisted in drafting the manuscript. All authors have read and approved the final manuscript.
